# Pregnancy Risk Perception and Associated Factors among Pregnant Women Attending Antenatal Care at Health Centers in Jabi Tehnan District, Amhara, Northwestern Ethiopia, 2021

**DOI:** 10.1155/2022/6847867

**Published:** 2022-12-13

**Authors:** Demeke Andebet Alemu, Ambaye Minayehu Zegeye, Liknaw Bewket Zeleke, Wale Kumlachew Dessie, Yilkal Dagnaw Melese, Yaregal Desselaw Tarik, Fentahun Tamene Zeleke, Dawit Misganaw Belay, Alemitu Ayele Siyoum, Berhane Teklay Asfaha

**Affiliations:** ^1^Hailu Alemu College, Department of Midwifery, Gojjam, Ethiopia; ^2^Department of Midwifery, College of Health Sciences, Assosa University, Assosa, Ethiopia; ^3^Debre Markos University, College of Health Sciences, Department of Midwifery, Debre Markos, Ethiopia; ^4^Department of Nursing, College of Health Sciences, Assosa University, Assosa, Ethiopia; ^5^Wolkite University, College of Health Sciences, Department of Midwifery, Wolkite, Ethiopia; ^6^Madawalabu University, College of Health Sciences, Department of Midwifery, Madawalabu, Ethiopia

## Abstract

**Background:**

Pregnancy risk perception affects a pregnant woman's decision about health care services such as prenatal care, place of birth, choices about medical interventions, adherence to medical procedures, and recommendations. Therefore, the study is aimed at assessing pregnancy risk perception and associated factors among pregnant women attending antenatal care at health centers in Jabi Tehnan District.

**Methods:**

An institutional-based cross-sectional study was conducted among 424 mothers attending ANC at health centers in the Jabi Tehnan District from April 1 to 30, 2021. Data was collected through a face-to-face interview using a structured questionnaire which was developed according to the health belief model. The logistic regression model was used using an adjusted odds ratio with 95% CI and *p*value < 0.05 to declare significance and associations.

**Result:**

Four hundred twenty four (424) pregnant women were interviewed of which nearly half of the respondents 48% (43.2%, 52.7%) had good pregnancy risk perception. Women who had a history of obstetric complications (AOR: 95% CI = 3.44 : 1.73, 6.83), those who knew at least one pregnancy danger sign (AOR: 95% CI = 5.22, 2.46, 11.07), pregnant women who had a bad obstetric history (AOR: 95% CI = 2.23 : 1.13, 4.41), and knowing women who died due to pregnancy-related complications (AOR: 95% CI = 2.85 : 1.45, 5.60) were more likely to have good perception towards pregnancy risk compared to their counterparts.

**Conclusion:**

Obstetric complications, awareness of pregnancy danger signs, bad obstetric history, and known women who died due to pregnancy-related complications were found to be significantly associated with pregnancy risk perception.

## 1. Background

Risk perception means an individual's expectation about the probability, characteristics, and severity of an event, and it is an important construction for different health behavior theories such as the health belief model, protection motivation theory, and prospect theory [1]. An individual making a judgment about the probability of an event is based on experience [2].

A greater perception of health risks leads to conducting protective action. Therefore, it is important to understand the perception of health risks of pregnancy and the accuracy of those perceptions [3]. Pregnancy risk perception affects a pregnant woman's decision about health care services like prenatal care, place of birth, choices about medical interventions, adherence to medical procedures and recommendations, and health behaviors [4–6].

Pregnancy is a normal physiological process, but some of the common discomforts of pregnancy may make the pregnant woman feel ill. Ranging from mildly irritating to life-threatening conditions [7], pregnancy and childbirth are often perceived as normal life events without justification in many developing countries, including Ethiopia. Obstetric complications are high among normal pregnancies. These complications occurred following warning signs called obstetric danger signs [8–11].

The top commonly manifested danger signs during labor and childbirth are severe vaginal bleeding, prolonged labor, convulsions, and retained placenta. Besides, dangerous signs might occur during the postnatal period, which include severe vaginal bleeding, unconsciousness, and fever [12, 13]. Every pregnant woman is at risk of facing pregnancy-related complications that could end in death or injury to both herself and the newborn [14]. From pregnancy-related complications, hemorrhage, obstructed labor, pregnancy-induced hypertension, puerperal sepsis, and unsafe abortion are the five leading causes of maternal death in Ethiopia from 1990 to 2016. Early detection and management of those complications are important to reduce maternal mortality [15].

Pregnancy and childbirth complications are major causes of maternal death. Complications that develop during pregnancy account for 72.5% of maternal deaths are called direct causes of maternal death. Globally, the magnitudes of direct cause's maternal death are hemorrhage (27.1%), pregnancy-induced hypertension (14%), puerperal sepsis (10.7%), and unsafe abortion (7.9%). In Ethiopia, hemorrhage (29.9%), obstructed labor (22.34%), pregnancy-induced hypertension (16.9%), puerperal sepsis (14.68%), and unsafe abortion (8.6%) are major causes of maternal death [15–17].

Due to obstetric complications globally every day, 810 women died. The developing countries accounted for 94% (277,300) of these deaths. Of these eighty-six percent of deaths are occurred in both Sub-Saharan Africa and Southern. Sub-Saharan Africa has the highest maternal mortality ratio at 66.44% (196 000) of maternal deaths annually. In Ethiopia, the maternal mortality ratio is significantly reduced from 1030 deaths per 100,000 live births (2000) to 401 death per 100,000 live births (2017), but it remains high [18].

The maternal mortality ratio in developing countries in 2015 is twenty times higher than in developed countries. The difference in maternal mortality ratio is also present within countries between urban and rural residents and between high and low-income women [19].

Pregnancy and childbirth-related mortality are unavoidable due to three delays. These are delays in deciding to seek care, delays in accessing and reaching appropriate care, and delays in receiving appropriate care once a health facility is reached. The first delay influences the probability of the second and third delay. Their poor perception of pregnancy-related risks and complications leads pregnant women to delay decision-making to seek obstetric care [20, 21].

Therefore, this study is intended to determine pregnancy risk perception and associated factors among pregnant women attending antenatal care at health centers in Jabi Tehnan District, Amhara, Northwest Ethiopia, 2021.

## 2. Methods

### 2.1. Study Area and Period

The study was conducted in Jabi Tehnan District, West Gojjam Zone, Amhara Region, Northwest Ethiopia. Jabi Tenhnan is bordered on the southwest by Dembech, on the west by Bure, on the northwest by Sekela, on the north by Kuarit, and on the east of Degadamot district. It is located at 387 km from Addis Ababa in the northwest part of Ethiopia. According to the report from the district in 2016, it has 39 kebeles with a total population of 218,447 and 125,323 adults. In the district, there are 11 health centers and 39 health posts. The health centers give different clinical services such as family planning, antenatal care, delivery, and testing of HIV for the nearby community. All health centers provide ANC services for the nearby community. The majority of the inhabitants practiced Orthodox Christianity (97.96%) while 2.02% were Muslim [22, 23]. This study was conducted from April 01, 2021 to April 30, 2021.

### 2.2. Study Design

Institution-based cross-sectional study design was employed.

### 2.3. Source and Study Population

The source population of this study was all pregnant women coming to antenatal care service in Jabi Tehnan District health centers, and the study population was all pregnant women attending to antenatal care service in Jabi Tehnan District health centers during the data collection period.

### 2.4. Sample Size Determination

The sample size was calculated using the single population proportion formula by considering 50% of the population has good pregnancy risk perception. The size of the sample was calculated as follows:

(1) $$\begin{array}{c} n=\frac{Z^{2}\alpha /2\times \left(p\left(1-p\right)\right)}{d^{2}}. \end{array}$$

- *n* was the sample size
- Z^2^*α*/2 was the 95% confidence interval (standard value of 1.96)
- *P* was the estimated proportion of patients with good pregnancy risk perception = 50%
- *d* is the margin of error (precision error) = ±5%

(2) $$\begin{array}{c} n=\frac{\left(1.96\right)2\times 0.5\times 0.5}{\left(0.05\right)2}=385. \end{array}$$

Then, by adding a 10% nonresponse rate, the total sample size was 424.

### 2.5. Sampling Technique and Procedure

**Figure 1:**
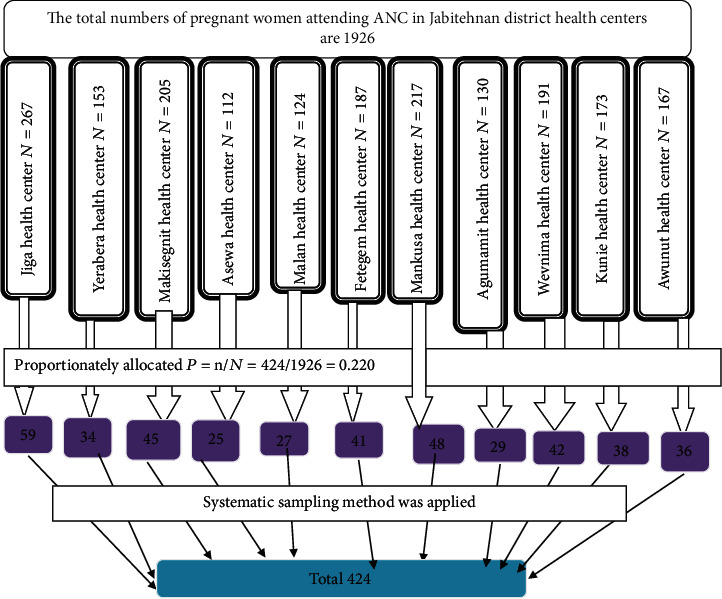
Sampling procedure to determine pregnancy risk perception and associated factors among pregnant women attending antenatal care at health centers in Jabi Tehnan District, Amhara, Northwest Ethiopia, 2021.

There are eleven health centers in the Jabi Tehnan District. All health centers were included in the study, and a systematic sampling technique was used to collect data. The total number of pregnant women attending ANC per month for the previous three consecutive months in each health center was taken from the ANC tally record book. The average number of pregnant women attending ANC per month was calculated. Based on the total number of pregnant women attending antenatal care in Jabi Tehnan District health center, the total sample was divided to each health center proportionally. *p* = *n*/*N*, *p* times of total antenatal care attendants per month in each health center (Figure 1).

### 2.6. Inclusion and Exclusion

#### 2.6.1. Inclusion Criteria

Pregnant women coming to antenatal care service in Jabi Tehnan District health center and no exclusion.

### 2.7. Study Variables

#### 2.7.1. Dependent Variables

Pregnancy risk perception (poor/good).

#### 2.7.2. Independent Variables

- Sociodemographic factors (age, marital status, education, occupation, religion, place of residence, income, and partner education)
- Knowing a woman who died due to pregnancy-related complications
- Obstetric factors (gravidity, parity, history of obstetric complications, pregnancy danger signs, number of ANC visits, and bad obstetric history)
- Knowledge of pregnancy danger signs

#### 2.7.3. Operational Definition

Pregnancy risk perception means respondents' perception of their risk of developing pregnancy-related complications and their perceived severity of consequences related to pregnancy and labor complications [24].

#### 2.7.4. Data Collection Tools

The tools were first prepared in English language and later translated into Amharic language and back to English language again to maintain its consistency and have four parts. These are the following indetails.

#### 2.7.5. Tool I Sociodemographic Characteristics

Sociodemographic characteristics such as age, marital status, education, working status, place of residence, income, partner education, and knowing a woman who died in pregnancy.

#### 2.7.6. Tool II Obstetric Characteristics

Obstetric characteristics such as total number of pregnancies, the total number of live birth, history of obstetric complications during a previous pregnancy or labor and delivery or postnatal period, history of current pregnancy danger signs, previous place of birth, and bad obstetric history.

#### 2.7.7. Tool III Knowledge of Pregnancy Danger Signs

The client's knowledge of pregnancy danger signs is assessed as a factor for their perception of pregnancy risk. Knowledge about pregnancy danger signs (yes or no options for being familiar with each pregnancy danger sign) is the source of information about the pregnancy danger sign. This tool consists of 10 questions, which focus on general knowledge of pregnancy danger signs.

#### 2.7.8. Tool IV Pregnancy Risk Perception Using the Health Belief Model

The client's pregnancy risk perception using health belief model constructs was assessed. Clients are asked a question about two constructs of the health belief model that ranges from five to twenty-five score for perceived susceptibility and seven to thirty-five score for perceived severity. The total scores were calculated from the combined questions ranging from twelve to sixty. The questions have five options (1 = strongly disagree, 2 = disagree, 3 = neutral, 4 = agree, and 5 = strongly agree) [25].

*(1) Data Collection Procedure*. Data were collected from April 1 to 30, 2021. Eleven midwives who are not working at the study site were recruited and trained for data collection. One day of training on the objective of the study, methods of data collection procedures, and tools of data collection for data collectors and supervisors were given. Each data collector completed questionnaires by interviewing clients who came on the day and completed them within 30 days. Regular supervision was done during data collection.

*(2) Data Quality Control Measuers*. To ensure the quality of data, the training was given on the objective of the study, methods of data collection procedures, and tools of data collection by the researcher. A pretest was conducted in 5% of the sample on March 2021 Finote Selam health center. Cronbach's alphas (0.89 up to 0.90) were used to check the internal consistency and reliability of the item. The necessary modification was made for any ambiguity, confusion, and difficult words based on pretest data analysis. Each data collector and supervisor were checked before and immediately after collection for the completeness and consistency of the questionnaire.

*(3) Data Processing and Analysis*. The data was cleaned, coded and entered, and analyzed using Statistical Package for Social Sciences (SPSS) version 20. Descriptive statistics such as frequency, percentage, standard deviation, and mean were used to characterize the participants in terms of sociodemographic variables, obstetric variables, and knowledge of pregnancy danger signs. A logistic regression model was fitted to assess the association between dependent and independent variables with a *p* value of ≤0.25 in the bivariable analysis that will be included in the multivariable analysis. The adjusted odds ratio together with 95% confidence intervals was computed, and the results with *p*value < 0.05 were considered to declare a result as significantly associated.

## 3. Results

### 3.1. Sociodemographic Characteristics

Four hundred twenty-one pregnant women have completed the questionnaire making the response rate of the study 99.3%. The mean age of respondents was 26.99 + 6.22, and nearly one-third (33.3%) of respondents were aged between 25 and 29 years. All respondents were Amhara in ethnicity, and 93.8% were orthodox in religion. Almost all the respondents (99.8%) were married, and more than half (58.7%) of the respondents were housewives. Of the total study participants, 57% of respondents lived in rural areas. Concerning educational status, slightly more than one-third (42.7%) of respondents have no formal schooling (Table 1).

### 3.2. Obstetric Characteristics

Of 421 respondents, 47.5% were primigravida, and 48% were nulliparous. Of multigravida women, 52% had experienced obstetric complications in the previous pregnancy or labor or postpartum period, and the majority (64.3%) of respondents had no history of bad obstetric history (Table 2).

### 3.3. Knowledge of Pregnancy Danger Signs

Vaginal bleeding (87.9%) was the most common mentioned danger sign during pregnancy followed by loss of fetal movement (53.2%), the onset of labor before the expected date of delivery (52.2%), and persistent vomiting (48.4%). The least mentioned danger signs during pregnancy were convulsion/loss of consciousness (27.1%) (Table 3).

### 3.4. Pregnancy Risk Perception

**Figure 2:**
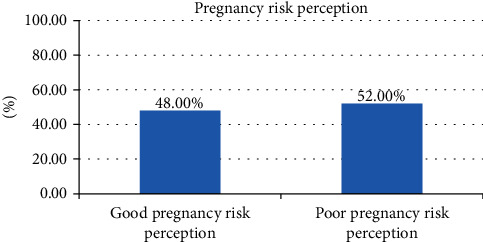
Perceived severity of pregnancy-related complications among pregnant women attending antenatal care at Jabi Tenhan District health centers in April 2021 (*n* = 421).

Of the total respondents, nearly half of them, 48% (43.2% and 52.7%), had good pregnancy risk perception as shown in (Figure 2).

Concerning the perceived severity of pregnancy-related complications, 57.9% perceived that pregnancy and delivery problems would last a long time while 55.9% of respondents perceived that pregnancy complications would not threaten the relationship with their partner. The majority of the respondents (74.3%) perceived that their baby will be born prematurely. 32.8% of respondents strongly agree that their baby would not survive the pressure that comes with labor and delivery (Table 4).

### 3.5. Factors Affecting Pregnancy Risk Perception in Jabi Tehnan

In bivariable analysis, residence, own income, knowing a woman who died due to pregnancy-related complications, past obstetric complications, current pregnancy danger signs, the number of ANC visits, bad obstetric history, and awareness of pregnancy danger signs showed *p* value less than 0.25 making them eligible for multivariable analysis.

In multivariable analysis awareness of pregnancy danger signs, bad obstetric history, past obstetric complications, and knowing a woman who died due to pregnancy-related complications were associated with pregnancy risk perception. Those who know at least one pregnancy danger sign were 5.2 times (AOR: 95% CI = 5.22 : 2.46, 11.07) more likely to have good pregnancy risk perception than their counterparts. Respondents who had bad obstetric history were 2.2 times (AOR: 95% CI = 2.23 : 1.13, 4.41) more likely to have good pregnancy risk perception than their counterparts.

Respondents who had past obstetric complications were 3.44 times (AOR: 95% CI = 3.44 : 1.73, 6.83) more likely to have good pregnancy risk perception than their counterparts. Knowing women died due to pregnancy-related complications were 2.85 times (AOR: 95% CI = 2.85 : 1.45, 5.60) more likely to have good pregnancy risk perception than their counterparts (Table 5).

## 4. Discussion

A major finding of this study was that above half of pregnant women had significantly poor risk perception. Out of the total study subjects, 48% had a good pregnancy risk perception. The independent variables that affect pregnancy risk perception were history of obstetric complications, knowing women who died due to pregnancy-related complications, bad obstetric history, and awareness of pregnancy danger signs.

In this study, 48% of pregnant women had a good pregnancy risk perception. Studies conducted at health and medical enters of Hamadan City in the west of Iran, 2 tertiary-care hospitals in Winnipeg, Manitoba, two major teaching hospitals of a city in Western Canada, and urban tertiary care hospital in western Canada showed that their mean score perception of pregnancy risk was below the midpoint of scales. It means that 100% of their study participants perceived that their susceptibility to pregnancy-related complications was mild. This difference might be due to that all the research was conducted using a visual analog scale tool measurement which is only administered for literate people, data collection tool difference, sociocultural difference, sample size difference, and time gap of the study [26–29].

This study showed that 48.9% (43.9% and 53.7%) of study subjects perceived that they were susceptible to pregnancy-related complications. This finding was higher than those studies conducted in Mandera County, Kenya (14.5%). This difference might be due to sociocultural differences of study participants, study population, and time gap of the study [25].

This research showed that 29.7% (25.7% and 34.4%) of pregnant women were perceived them to be susceptible to bad pregnancy outcomes, and 58.2% (53.7% and 62.9%) were perceived that they are susceptible to difficult pregnancy periods. This finding was consistent with the study conducted in Mandera County, Kenya (28.2%) [25].

In this study, 74.3% (70.5% and 78.4%) of pregnant women perceived that their baby will be born prematurely. This result was consistent with studies conducted in Mandera County, Kenya (75%). But studies conducted at Hamadan City, Iran and Winnipeg, Manitoba showed that mean score perceptions were below the midpoint of scales that indicate mild risk perception. This difference might be due to the difference of measurement scale, sociocultural difference, and time gap of the study [25, 26, 28].

This study revealed that 45.1% (40.1% and 49.9%) of pregnant women were perceived that the occurrence of pregnancy complications would not threaten their relationship with their partners, which is lower than a study conducted in Mandera County, Kenya (51%). This difference might be due to the sociocultural difference of study participants and the time gap of the study. Besides, it may be because Ethiopian women were honest with their husbands to keep their promise during the marriage [25].

In this study, mean score perception pregnancy risk in pregnant women showed that they did not perceive that they are at risk for dying due to pregnancy-related complications. Studies conducted in Winnipeg, Manitoba, Western Canada, and the west of Iran had mild risk perception. This difference may be due to differences in measurement scale, data collection tool, sociocultural difference, and educational status of respondents [26–28].

Approximately, seventy percent of women perceived that their babies would die during labor and delivery. This result was inconsistent with studies conducted in western Canada and Hamadan City in the west of Iran. This difference may be due to differences in measurement scale, sociocultural difference, and educational status of respondents [26, 27].

Pregnant women who had awareness of pregnancy danger signs were more likely to have positive pregnancy risk perception than their counterparts. This may be due to knowledge about pregnancy danger signs clearing rumors about pregnancy-related complications and increasing their awareness about pregnancy-related complications.

According to this study, pregnant women with bad obstetric history were more likely to have positive pregnancy risk perception. This may be due to pregnant women being familiarized with the bad obstetric outcome and learning their susceptibility towards pregnancy-related complications.

In this research, one of the factors that affecting positively, pregnancy risk perception was knowing a woman who died due to pregnancy-related complications. This may be because these women learned the probability of developing pregnancy-related complications as well as the consequences of pregnancy-related complications from women's death due to pregnancy-related complications.

Generally, pregnant women with a bad obstetric history, past obstetric complication, and knew a woman who died due to pregnancy-related complications were associated with pregnancy risk perception. This result was similar to the concept of availability of the Heuristic approach which means that an individual makes a judgment about likelihood of an event based on experience or information from others [2]. In this study, experience and information from others are pregnant women with bad obstetric history and past obstetric complications and knew a woman who died due to pregnancy-related complications, respectively.

In this study, age of pregnant women is not significantly associated with pregnancy risk perception. But women aged < 18 years had statistically significantly associated with pregnancy risk perception than women aged 18 to 35 in studies conducted at health and medical centers of Hamadan City in the west of Iran [26]. This difference might be due to sampling size difference (421 pregnant women versus 240 pregnant women), age category difference (15-43 years versus <35 years), study participant difference (both nulliparous and multiparous versus only nulliparous), measurement scale differences (Likert scale versus visual analog scale), and the difference in data collection tools.

## 5. Conclusion

The pregnant women perceive that they were risky in developing pregnancy-related complications, and their consequences were low. Only forty-eight percent of pregnant women had good pregnancy risk perception. This is likely to have implications for medical care and pregnancy outcomes.

History of obstetric complication and bad obstetric history was associated with a higher degree of actual risk perception in pregnancy whilst women who knew the death of pregnant women due to pregnancy complication and knew at least one pregnancy danger signs were more likely to be concerned about risk.

### 5.1. Strength

The strength of this research is that it is the primary research conducted in Ethiopia and Africa. Besides, the research measures the outcome variables using the two components of the health belief model (perceived susceptibility and severity).

### 5.2. Limitations

This study was conducted within the clinical area. Therefore, the results cannot be generalized to a woman who failed to attend antenatal care. In addition, cultural influence of pregnancy risk perception should be identify using a qualitative research approach, but this research lacks it.

## Figures and Tables

**Table 1:** Sociodemographic characteristics of pregnant women attending antenatal care at Jabi Tenhan District health centers in April 2021 (*n* = 421).

Variables	Category	Frequency	Percent
Age	15-19	47	11.1
	20-24	115	27.3
	25-29	140	33.3
	30-34	57	13.5
	35-39	42	10
	>39	20	4.8

Religion	Orthodox	395	93.8
	Muslim	26	6.2

Marital status	Married	420	99.8
	Divorced	1	0.2

Occupation	Housewife	247	58.7
	Employed	66	15.7
	Merchant	74	17.6
	Student	22	5.1
	Daily labor	12	2.9

Residence	Urban	181	43
	Rural	240	57

HH income	Yes	140	33.3
	No	281	66.7

Educational status	Illiterate	180	42.7
	Primary school	85	20.2
	Secondary school	93	22.1
	College/university	63	15

Partner educational level	Illiterate	233	55.3
	Primary school	36	8.6
	Secondary school	50	11.9
	College/university	102	24.2

**Table 2:** Obstetric characteristics of pregnant women attending antenatal care at Jabi Tenhan District health centers in April 2021 (*n* = 421).

Variable	Category	Frequency	Percent
Gravidity	Primigravida	200	47.5
	Multigravida	169	40.1
	Grand multigravida	52	12.4

Parity	Null	202	48
	One	77	18.3
	Two up to four	112	26.6
	Five and above	30	7.1

Past obstetric complications	Yes	115	52
	No	106	48

Pregnancy danger signs	Yes	154	36.6
	No	267	63.4

Bad obstetric history	Yes	79	35.7
	No	142	64.3

**Table 3:** Knowledge of pregnancy danger signs among pregnant women attending antenatal care at Jabi Tehnan District health centers in April 2021 (*n* = 314).

Variables	Frequency (%)	
Awareness of pregnancy danger signs	Yes	No
Vaginal bleeding	276 (87.9%)	38 (12.1%)
Severe headache	126 (40.1%)	188 (59.9%)
Persistent vomiting	152 (48.4%)	162 (51.6%)
Swollen hand/face	146 (46.5%)	168 (53.5%)
Severe abdominal pain	100 (31.8%)	214 (68.2%)
Convulsion/loss of consciousness	85 (27.1%)	229 (72.9%)
Blurred vision/dizziness	148 (47.1%)	166 (52.9%)
Loss of fetal movement	167 (53.2%)	147 (46.8%)
Water break before labor	141 (44.9%)	173 (55.1%)
The onset of labor before the expected date of delivery	164 (52.2%)	150 (47.8%)
Persistent fever	138 (43.9%)	176 (56.1%)

**(a):** 

Variables	Frequency (%)					Mean (SD)
(1) Perceived susceptibility	Strongly disagree	Disagree	Neutral	Agree	Strongly agree	
Getting extremely pregnancy-related complications	105 (24.9%)	41 (9.7%)	69 (16.5%)	171 (40.6%)	35 (8.3%)	2.98 (1.36)
Fears of having a difficult pregnancy period	71 (16.9%)	58 (13.7%)	47 (11.2%)	193 (45.8%)	52 (12.4%)	3.23 (1.31)
Good possibility to get complications related to delivery and the postpartum period	62 (14.7%)	55 (13.1%)	51 (12.1%)	163 (38.7%)	90 (21.4%)	3.39 (1.35)
Getting pregnancy-related complications are great	84 (19.9%)	99 (23.5%)	56 (13.3%)	132 (31.4%)	50 (11.9%)	2.92 (1.35)
Get bad pregnancy outcome	119 (28.3%)	78 (18.5%)	99 (23.5%)	100 (23.8%)	25 (5.9%)	2.61 (1.28)

**(b):** 

Variables	Frequency (%)					Mean (SD)
(2)Perceived severity	Strongly disagree	Disagree	Neutral	Agree	Strongly agree	
Pregnancy and delivery problems would last a long time	33 (7.8%)	44 (10.5%)	102 (24.2%)	167 (39.7%)	75 (17.8%)	3.49 (1.14)
Pregnancy complications would threaten the relationship with the partner	101 (24%)	57 (13.5%)	73 (17.3%)	103 (24.5%)	87 (20.7%)	3.04 (1.47)
Pregnancy-related complications can lead to permanent changes in life	46 (10.9%)	52 (12.4%)	64 (15.2%)	153 (36.3%)	106 (25.2%)	3.52 (1.29)
Pregnancy would not last to term	50 (11.9)	32 (7.6%)	56 (13.3%)	161 (38.2%)	122 (29%)	3.65 (1.30)
The baby would not survive the pressure that comes with labor and delivery	45 (10.7%)	47 (11.2%)	29 (6.9%)	162 (38.4%)	138 (32.8%)	3.72 (1.31)
If I got pregnancy-related complications, I fear I will not survive them	88 (20.9%)	61 (14.5%)	40 (9.5%)	128 (30.4%)	104 (24.7%)	3.24 (1.49)
Premature birth	36 (8.6%)	33 (7.8%)	39 (9.3%)	145 (34.4%)	168 (39.9%)	3.89 (1.25)

**Table 5:** Factors affecting pregnancy risk perception of pregnant women attending antenatal care at Jabi Tenhan District health centers in April 2021 (n = 421).

Variables		Pregnancy risk perception		COR (95% CI)	AOR (95% CI)	*p* value
		Good	Poor			
Knowing at least one pregnancy danger sign	Yes	166	148	2.21 (1.39, 3.49)	5.22 (2.46,11.07)	≤0.001
	No	36	71	1	1	

Bad obstetric history	Yes	54	25	2.49 (1.40, 4.43)	2.23 (1.13, 4.41)	0.022
	No	66	76	1	1	

Obstetric complication	Yes	78	37	3.21 (1.85, 5.58)	3.44 (1.73, 6.83)	≤0.001
	No	42	64	1	1	

Having pregnancy danger signs	Yes	107	47	4.12 (2.69, 6.30)	1.78 (0.87, 3.65)	0.116
	No	95	172	1	1	

Knowing women died due to pregnancy-related complications	Yes	129	75	3.39 (2.27, 5.06)	2.85 (1.45, 5.60)	0.002
	No	73	144	1	1	

Level of ANC visit	Fourth	49	43	2.52 (1.33, 4.79)	2.29 (0.78, 6.74)	0.131
	Third	68	59	2.55 (1.39, 4.67)	0.95 (0.35, 2.57)	0.918
	Second	62	66	2.08 (1.14, 3.80)	0.64 (0.22, 1.81)	0.39
	First	23	51	1	1	

Residence	Urban	73	108	0.58 (0.39, 0.86)	0.29 (0.078, 1.11)	0.071
	Rural	129	111	1	1	

Own income	Yes	60	80	0.73 (0.49,1.10)	3.52 (0.89, 13.80)	0.093
	No	142	139	1	1	

## Data Availability

The original data for this study are available from the corresponding author upon reasonable request.

## Abbreviations

ANC: Antenatal care
FANC: Focused antenatal care
HBM: Health belief model
MMR: Maternal mortality ratio
PPH: -Postpartum hemorrhage.
